# A new species group in *Megaselia*, the *lucifrons* group, with description of a new species (Diptera, Phoridae)

**DOI:** 10.3897/zookeys.512.9494

**Published:** 2015-07-06

**Authors:** Sibylle Häggqvist, Sven Olof Ulefors, Fredrik Ronquist

**Affiliations:** 1 Swedish Museum of Natural History, Department of Zoology, Box 50007, SE-10405 Stockholm, Sweden; 2 Stockholm University, Department of Zoology, Svante Arrhenius väg 18A, SE-10691 Stockholm, Sweden; 3 Färgerivägen 9, 38044 Alsterbro, Sweden; 4 Swedish Museum of Natural History, Department of Bioinformatics and Genetics, Box 50007, SE-10405 Stockholm, Sweden

**Keywords:** Phoridae, new species, *Megaselia albalucifrons* sp. n., *Megaselia lucifrons*, *Megaselia subnitida*

## Abstract

With 1,400 described species, *Megaselia* is one of the most species-rich genera in the animal kingdom, and at the same time one of the least studied. An important obstacle to taxonomic progress is the lack of knowledge concerning the phylogenetic structure within the genus. Classification of Megaselia at the level of subgenus is incomplete although Schmitz addressed several groups of species in a series of monographs published from 1956 to 1981. Another problem is the lack of molecular phylogenetic analyses to support morphology-based conclusions. As a contribution towards addressing these problems, we here circumscribe a previously unrecognized monophyletic lineage of *Megaselia* consisting of species similar to *Megaselia lucifrons*. We base this taxonomic decision on morphological study of an extensive phorid material from Sweden, complemented by molecular analyses of select exemplars using two markers (COI and 28S). We name the clade the *lucifrons* species group, and show that it contains three distinct species. Our results also demonstrate that *Megaselia subnitida* Lundbeck, 1920, previously treated as a synonym of *Megaselia lucifrons* Schmitz, 1918, is a separate species, and we remove it from synonymy. The third species in the group was previously unknown; we describe it here as *Megaselia albalucifrons* **sp. n.**

## Introduction

The Phoridae (Diptera), or scuttle flies, are one of the most diverse families of flies. There are currently more than 4,000 described species (Brown et al. 2015), and experts hold that this may represent as little as 10% or less of the true diversity (Disney 2006, Bonet et al. 2011). Most of the genera within the family are well-defined monophyletic groups with small to moderate numbers of species, but they only account for a small fraction of the total diversity. *Megaselia* is by far the largest genus in the family, a truly impressive radiation. With approximately 1,400 described species so far (Disney 2006), and many more expected, it might be the largest genus in the animal kingdom. It is apparently a fairly recent radiation; the oldest confirmed specimens of *Megaselia* do not show up in the fossil record until approximately 23 Myr from Dominican amber (Brown 1999), whereas the oldest fossil specimens from stem-group Phoridae are approximately 100 Myr, from the Cretaceous (Grimaldi and Cumming 1999).

It is unclear at this point whether *Megaselia* is monophyletic with respect to all of the remaining genera in the family, and the sheer size of the genus makes it difficult to study its phylogenetic structure. Indeed, the lack of knowledge concerning higher-level relationships within *Megaselia* is probably the most important impediment to further taxonomic progress on the genus. Although Schmitz indicated which groups within *Megaselia* he recognized, as published in Lindner’s “Die Fliegen” (1956–1981), a complete revision of the genus has never been completed and his work remains unfinished. Schmitz divided the genus into two subgenera, *Megaselia* and *Aphiochaeta* (Schmitz, 1926–1927), based on the pubescence of the anepisternum: it is naked in the former but hairy in the latter. The subgenera were then divided into divisions (“Abteilungen”) and sometimes further into series. The divisions were described in the first publication in this series on *Megaselia* (Schmitz, 1956), which were followed by revisions of each division, with keys to the species and morphological descriptions of all species. Unfortunately, the subgenus *Megaselia* with division VI to VIII was never published. For division VI, there is a key to the species but no descriptions of them.

**Figure 1.**
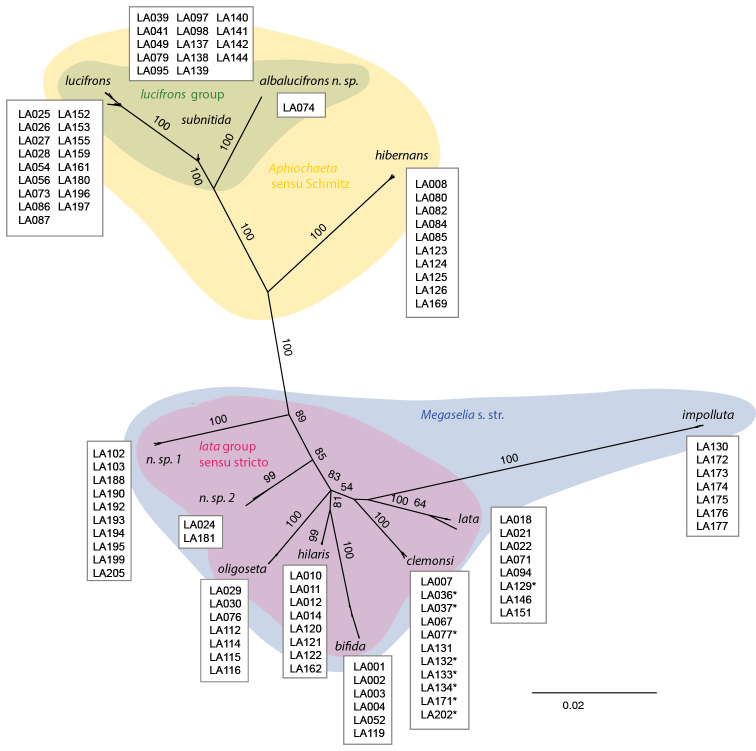
Majority rule consensus tree from a Bayesian phylogenetic analysis based on combined COI and 28S D2 data, showing that the *lata* group, as originally circumscribed by us, consists of two distinct but unrelated lineages that are consistent with the two subgenera *Megaselia* and *Aphiochaeta*: the *lata* group sensu stricto and the *lucifrons* group. Numbers on branches are posterior probabilities (PP, in percent). PP values are not given for branches inside the terminal clusters corresponding to species. The scale bar represents nucleotide substitutions per site. Numbers within boxes (LAXXX) are specimen IDs and indicate which specimens were used in the analysis. Specimens marked with asterisks (*) represent morphospecies that were not supported as distinct by the molecular analysis.

During our work on phorid material from the Swedish Malaise Trap Project (SMTP), a large-scale inventory of the Swedish insect fauna (Karlsson et al. 2005), we first identified a number of putative subgroups within *Megaselia* based mainly on morphological characters found in the male genitalia. We then selected one of these subgroups, the *lata* subgroup, for more detailed molecular study. The molecular results showed that the *lata* group, as originally circumscribed by us (sensu lato), consists of two unrelated lineages: the *lata* group sensu stricto and a monophyletic group of species similar to *Megaselia lucifrons* Schmitz, 1918 (Fig. 1).

**Figure 2.**
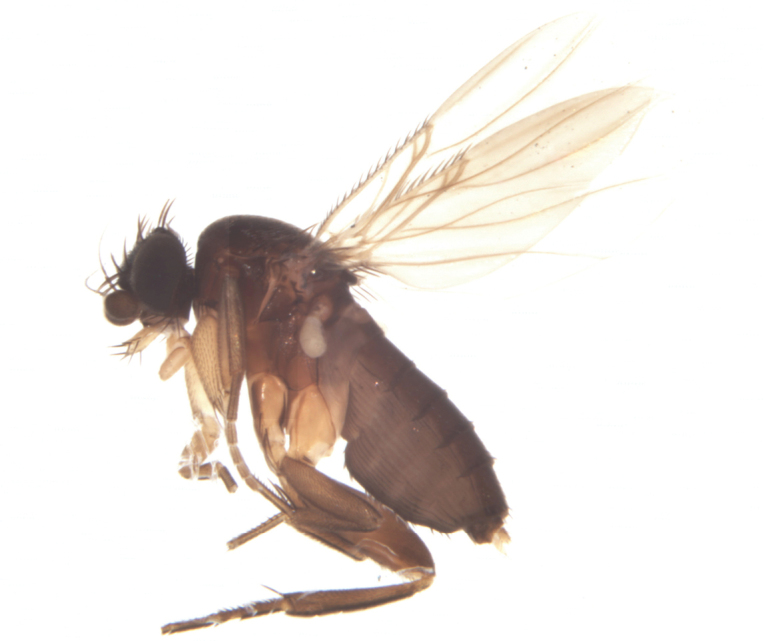
Lateral habitus of *Megaselia albalucifrons* sp. n.

In this paper, we focus on the latter group. We name this clade the *lucifrons* species-group and characterize it morphologically. Based on molecular and morphological data, we show that the group contains three species: *Megaselia lucifrons*; *Megaselia subnitida* Lundbeck, 1920; and *Megaselia albalucifrons* sp. n., which is described here (Fig. 2). We discuss the morphological features characteristic of the *lucifrons* group, give a key to the males, provide species diagnoses, and summarize what is known about their distribution and biology.

## Material and methods

### Material

The initial morphological studies were based on a large phorid material, mostly coming from the SMTP. The SMTP sampled the Swedish insect fauna using 75 Malaise traps run for three consecutive years (2003–2006) at 54 different sites, selected to represent a wide range of habitats (Karlsson et al. 2005). So far, approximately 35,000 phorid specimens from the SMTP material have been studied by SOU, with approximately 1,200 of them dissected and studied in detail. Critical characters in the latter specimens have been documented by drawings for future reference; these drawings are available upon request.

For the initial molecular analysis, we used 101 specimens from species representing the *lata* group, sensu lato, with *Megaselia impolluta* and *Megaselia hibernans* as outgroups. One of the lineages identified in this analysis, called the *lucifrons* group here, was selected for more detailed study. Specifically, 46 specimens of the *lucifrons* group were sequenced and 14 of these specimens were dissected. Based on the initial molecular analysis, we identified *Megaselia lata* (Wood, 1910) and *Megaselia hibernans* Schmitz, 1934 as suitable outgroups for the analyses of the *lucifrons* group.

Repositories of studied specimens and corresponding abbreviations are: Swedish Museum of Natural History (NHRS); Zoologisches Forschungsmuseum Alexander Koenig, Bonn, Germany (ZFMK); Natural History Museum of Denmark (ZMUC) and Natural History Museum of Los Angeles County, Los Angeles, USA (LACM). The holotype and paratypes of *Megaselia albalucifrons* are deposited in the collection of the Swedish Museum of Natural History.

### Morphological methods

**Figure 3.**
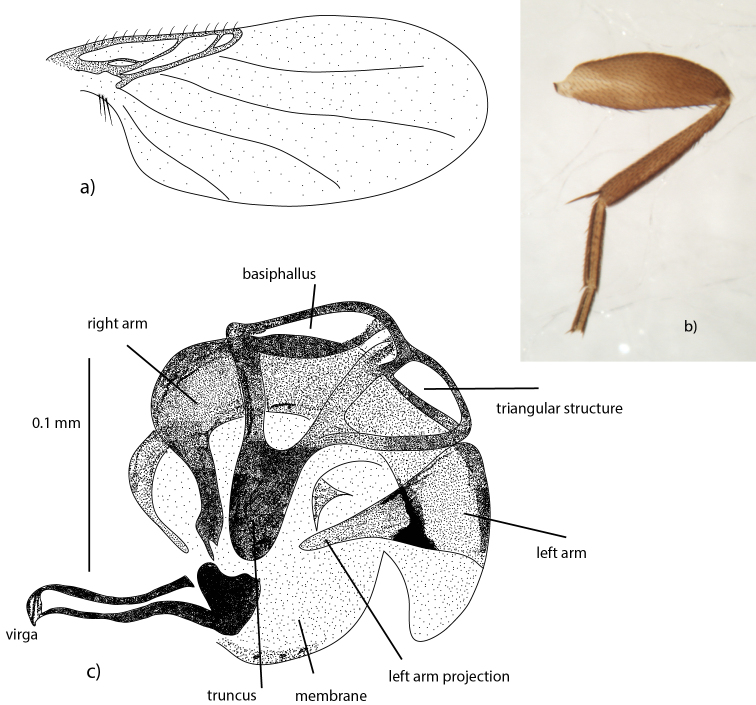
Wing (**a**), leg (**b**) and phallus in frontal view (**c**) of *Megaselia albalucifrons* sp. n.

Specimens were examined under a stereomicroscope with magnification up to 60×. Two specimens from each of the three species were dissected in order to examine different parts of the male genitalia (epandrium, hypandrium and phallus) more closely under a compound microscope with 400× magnification. Paper and pencil drawings were prepared using a camera lucida fitted to a compound microscope. The drawings were digitized and redrawn in Adobe Illustrator. Terminology of the male genitalia follows McAlpine et al. (1981) and Brown (1987) where homology of the structures is clear. Additional terminology is given in Figure 3c.

### Molecular methods

Genomic DNA was extracted using QIAGEN mini kit or GenMole robot. Two different DNA fragments were amplified and sequenced: the mitochondrial cytochrome c oxidase subunit I (COI) and the D2 variable expansion region of nuclear 28S rRNA (28S). The oligonucleotide primers used are LCO1490 and HCO2198 as listed in Folmer et al. (1994) for COI and D2F and D2R as listed in Sallum et al. (2002) for 28S. PCR amplifications were carried out in 25-µl volume using annealing temperatures between 50 and 55 °C.

Amplified products were checked on a mini-agarose-gel and purified using EXO1 and FastAP (EXOFAP). DNA sequencing was performed using the same primers as in the PCR reactions and the BigDyeTM Terminator ver. 3.1 Cycle Sequencing Kit (Applied Biosystems). Sequencing reactions were purified using the DyeEx 96 kit (QUIAGEN) and the samples were sequenced on an ABI Prism 3100 Genetic Analyzer (Applied Biosystems).

Sequence electropherograms were read and edited using Geneious Pro 5.3.4 (Biomatters Ltd.). Alignments for both genes were performed inside Geneious Pro 5.3.4 using the Consensus align option. All sequences were deposited in GenBank (Suppl. material 1).

### Ecological data

To study seasonal patterns in adult activity, the occurrence data for *Megaselia lucifrons* were recorded from the 24 individuals that were used in this study and an additional 268 SMTP specimens identified as *Megaselia lucifrons* by SOU. The collection dates (Malaise trap emptying dates) were pooled into 10.5 days long periods. We did not have enough data for the other two species to permit similar analyses.

### Data analyses

Genetic distances and numbers of parsimony informative characters were obtained using MEGA version 6 (Tamura et al. 2013). Phylogenetic analyses were conducted using MrBayes 3.2.2 (Ronquist et al. 2012), under default settings unless noted otherwise. Rather than selecting a nucleotide substitution model by *a priori* model selection, we used the “lset nst = mixed” option to integrate over substitution model space (Huelsenbeck et al. 2004). To model rate variation across sites, we used a gamma distribution with a proportion of invariable sites. The two genes were analyzed together for all taxa with COI data present in a combined analysis and separately. In the combined analysis, we used three data partitions: first and second codon positions in COI, third codon positions in COI, and 28S. A partition-specific rate multiplier was used to model rate variation among partitions. The separate COI and 28S analyses used the same model settings as the combined analysis, where applicable. The combined analysis was run for 30 million generation, the separate COI analysis for 6 million generations and the 28S analysis for 5 million generations. All analyses were conducted in two parallel runs until the average standard deviations of split frequencies reached 0.01 or below and the trace plots indicated good mixing. The first 25% of the generated trees was discarded as burnin. Data sets, MrBayes commands and resulting trees are available from TreeBase (http://purl.org/phylo/treebase/phylows/study/TB2:S17025) and as supplementary material.

Species circumscription was further analyzed using the program BP&P (Rannala and Yang 2003; Yang and Rannala 2010). This method uses multiple markers and accommodates incomplete lineage sorting due to ancestral polymorphism when inferring species circumscriptions. A gamma prior *G*(2, 1000), with mean 2/1000 = 0.002, was used for the population size parameters (qs). The age of the root in the species tree (t_0_) was assigned a gamma prior *G*(2, 1000), while the other divergence time parameters were assigned a Dirichlet prior (Yang and Rannala 2010: equation 2). Two species-delimitation algorithms were used (algorithm 0 with e = 2 and algorithm 1 with a = 2, m = 1).

## Results

### *Megaselia* species groups

To this date, we have identified approximately 65 species groups within *Megaselia* based on morphological characters. Some of these groups, such as Kryophile, Crassitarsale and Simplicitarsale have already been described by Schmitz and others, but most are new, even though they sometimes form subgroups of more familiar groups, such as the *pulicaria* complex (e.g. Disney 1999).

The *lata* group, as originally circumscribed by us (sensu lato), is characterized morphologically by the large and spinose labellum, the long costal vein that is about half the length of the wing, the long first costal sector, the short costal cilia and the strong proctiger hair. In the phallus, the base of the left arm projection is clearly sclerotized. These characters are not unique to the *lata* group, except possibly the sclerotized base of the left arm of the phallus, but the combination is unique and not found in other members of *Megaselia*.

Defined based on this suite of morphological characters, the *lata* group diagnosis cuts across the subgenera *Megaselia* and *Aphiochaeta* recognized by Schmitz (1926–1927), that is, it includes both species with naked and those with pilose anepisternum. However, our initial molecular analysis (Fig. 1) indicates that the *lata* suite of morphological characters arose at least twice, once in each subgenus recognized by Schmitz. As a consequence, we divide the initial *lata* group into two: the *lata* group, sensu strico, belonging to the subgenus *Megaselia* sensu stricto, and the group named here the *lucifrons* group, belonging to the subgenus *Aphiochaeta*.

Further studies of the *lata* suite of morphological characters indicated that there is a difference between the *lata* group, sensu stricto, and the *lucifrons* group in the left arm of the phallus. The left arm projection of the phallus is found in species of the *Megaselia lucifrons* group but not in species of the *Megaselia lata* group. Males of the *lucifrons* group (except *Megaselia subnitida*) also have a light patch on the hind femur, which is absent in males of the *lata* group, sensu stricto. If the absence in *Megaselia subnitida* is secondary, this may also be a good character distinguishing the groups.

### Phylogenetic analysis of the *lucifrons* group

In total, we analyzed phylogenetic relationships among 48 specimens belonging to the *lucifrons* group or one of the two outgroup species, *Megaselia lata* and *Megaselia hibernans*. Across the entire dataset, the COI alignment (658 bp) had 116 variable nucleotide positions, 52 of which were parsimony informative. The 28S alignment (518 bp) contained 21 variable nucleotide positions, 10 of which were parsimony informative.

**Figure 4.**
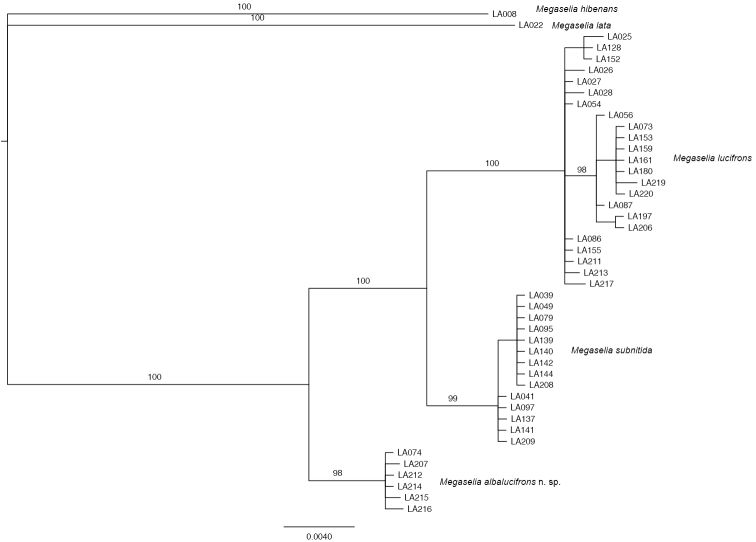
Majority rule consensus tree from a Bayesian analysis of *lucifrons* group relationships based on COI and 28S D2 data. *Megaselia lata* and *Megaselia hibernans* were used as outgroups. Numbers on branches are posterior probabilities (PP, in percent). PP values are not given for branches inside the terminal clusters corresponding to species. The scale bar represents nucleotide substitutions per site.

In the Bayesian phylogenetic analysis of the combined data, the *lucifrons* group was monophyletic with respect to the included outgroups with a posterior probability (PP) of 100% (Fig. 4). The sequenced individuals of the *lucifrons* group clearly clustered into three groups, identified based on the literature and study of type specimens as the species *Megaselia lucifrons*, *Megaselia subnitida*, and *Megaselia albalucifrons* sp. n..

Species monophyly was well supported (PP 98–100%). Intraspecific genetic distances in COI were 1–2%, while interspecific differences varied from 14 to 21%. The analyses also clearly indicated that *Megaselia lucifrons* and *Megaselia subnitida* are more closely related to each other than to *Megaselia albalucifrons*. The results from the BP&P analysis indicate that the three species of the *lucifrons* group are separate species, with a posterior probability of 0.93 to 0.94 for delimiting five species (two outgroup species in addition to the three species of the *lucifrons* group). The results from the separate COI analysis were very similar to the results from the combined analysis, whereas the separate 28S analysis results were less well resolved but not in conflict with the COI results.

### Morphological and biological variation in the *lucifrons* group

The morphometric analyses revealed that *Megaselia subnitida* is, on average, a larger species than the other two, with longer wings relative to the body length (Table 1). In addition, there are some minor morphometric differences among the species in the wing venation. The coloration of the hind femur differs among males of the tree species: *Megaselia albalucifrons* and *Megaselia lucifrons* both have a light patch basally, while this patch is absent in *Megaselia subnitida*.

**Table 1.** Measurements of body length, frons width and height, wing length and lengths of the costal sectors in mm for males of the three species. Costal sector ratio is given as the ratio of all three costal sectors divided by costal sector 3. Numbers in parentheses behind species name indicate the number of individuals measured.

Species	*Megaselia albalucifrons* (6)	*Megaselia lucifrons* (27)	*Megaselia subnitida* (18)
Body length	1.65 ± 0.09	1.58 ± 0.08	1.78 ± 0.12
Frontal width/height	1.05 ± 0.06	1.08 ± 0.04	1.16 ± 0.06
Wing length	1.41 ± 0.11	1.38 ± 0.09	1.66 ± 0.07
Costal lenth	0.61 ± 0.06	0.62 ± 0.03	0.75 ± 0.06
Costal sector 1	0.40 ± 0.04	0.43 ± 0.03	0.51 ± 0.06
Costal sector 2	0.13 ± 0.03	0.11 ± 0.02	0.16 ± 0.02
Costal sector 3	0.09 ± 0.01	0.07 ± 0.01	0.09 ± 0.01
Costal sector ratio	4.44 : 1.44 : 1	6.14 : 1.57 : 1	5.67 : 1.78 : 1

**Figure 5.**
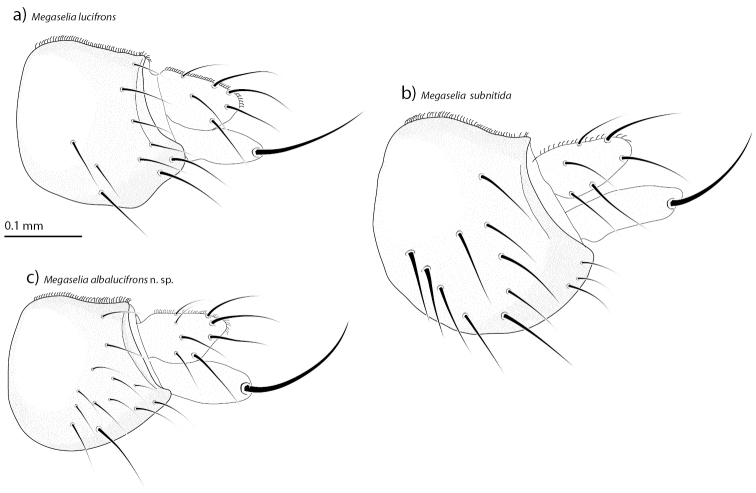
The left side of the epandria in lateral view of *Megaselia lucifrons* (**a**), *Megaselia subnitida* (**b**) and *Megaselia albalucifrons* sp. n. (**c**).

**Figure 6.**
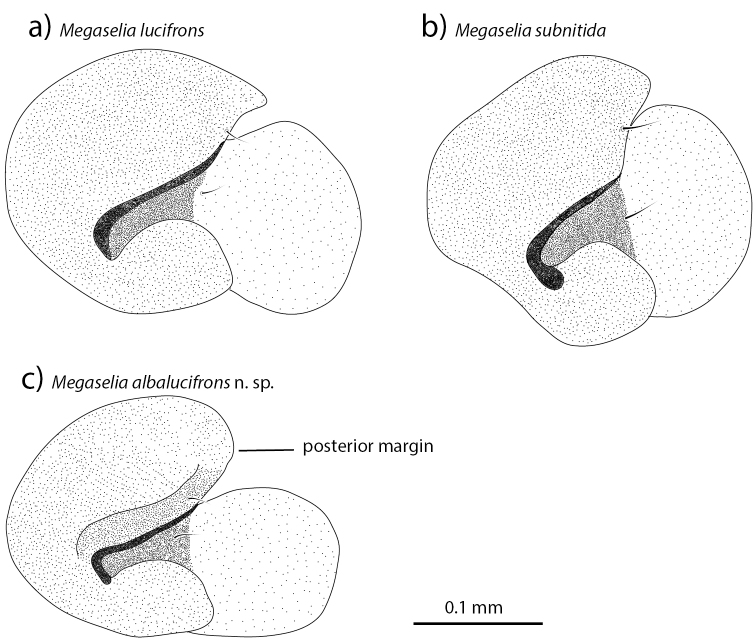
The hypandria in dorsal view of *Megaselia lucifrons* (**a**), *Megaselia subnitida* (**b**) and *Megaselia albalucifrons* sp. n. (**c**).

The male genitalia show a number of species specific differences. The entire epandrium in *Megaselia subnitida* is distinctly expanded ventrally (Fig. 5b). In *Megaselia lucifrons*, the ventral margin of the epandrium’s left side has a much smaller but distinct ventral expansion in the middle (Fig. 5a), while the margin is evenly rounded and lacks all ventral expansions in *Megaselia albalucifrons* sp. n. (Fig. 5c). Hairs on the lower part of the epandrium appear to be diagnostic for the species. *Megaselia lucifrons* has a few long hairs towards the proctiger (Fig. 5a), while the hairs in the same position in *Megaselia albalucifrons* and *Megaselia subnitida* are shorter. In all three species, one hair on the lower margin of the epandrium is elongated. In *Megaselia albalucifrons*, it is much longer than the surrounding hairs (Fig. 5c) while the difference is less dramatic in the other species. Furthermore, the cerci in *Megaselia lucifrons* are more pendulous than in *Megaselia albalucifrons* and *Megaselia subnitida*. *Megaselia subnitida* has a larger epandrium than the other species, both in absolute terms and relative to body size. In the hypandria of the three species, the shape of the left lobe is different among species (Fig. 6). Both *Megaselia lucifrons* and *Megaselia subnitida* have a broader lobe than *Megaselia albalucifrons*; the lobe is particularly short and broad in *Megaselia subnitida* (Fig. 6b). In both *Megaselia lucifrons* and *Megaselia subnitida*, the posterior margin of the right side of the hypandrium is concave while in *Megaselia albalucifrons* it is convex (Fig. 6). The shape of the phallus is similar in all species, with the base of the left arm projection being clearly sclerotized. Fig. 3c shows the phallus of *Megaselia albalucifrons*. In *Megaselia lucifrons* and *Megaselia subnitida*, the tip of the truncus is not as slim and rounded as in *Megaselia albalucifrons*; in *Megaselia lucifrons* it is almost formed as a quadrangular.

**Figure 7.**
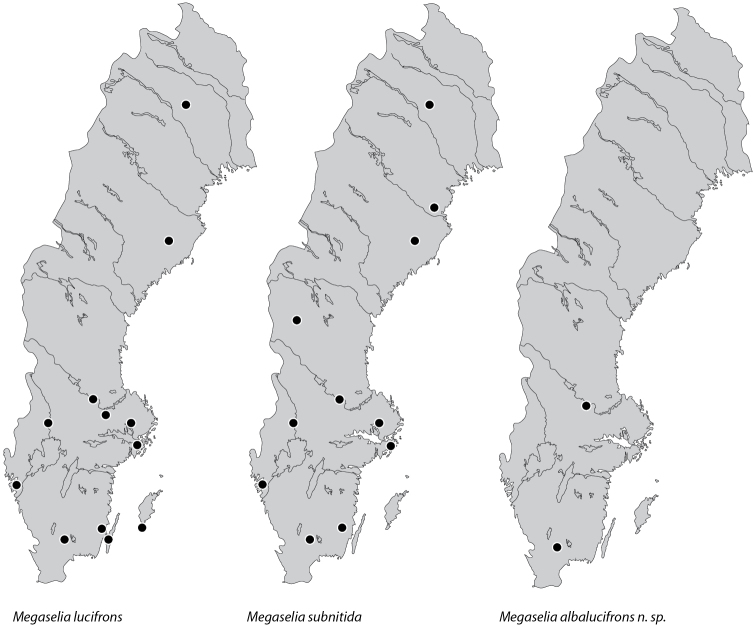
Map of Sweden showing the distribution of *Megaselia lucifrons*, *Megaselia subnitida* and *Megaselia albalucifrons* sp. n. Dots indicate the positions of the Malaise traps in which the species were found.

Both *Megaselia subnitida* and *Megaselia lucifrons* are common and widely distributed in Sweden (Fig. 7), even though only *Megaselia lucifrons* has so far been recorded from the eastern islands of Öland and Gotland in the Baltic Sea. *Megaselia albalucifrons* appears to be a less common and more locally distributed species; so far, it has only been recorded from two sites in the southern third of Sweden. It is clear that the *lucifrons* group is more widely distributed in Europe but many of the records are difficult to confirm as having the correct species determination, especially in the period after 1988 when *Megaselia subnitida* was considered a synonym of *Megaselia lucifrons*.

**Figure 8.**
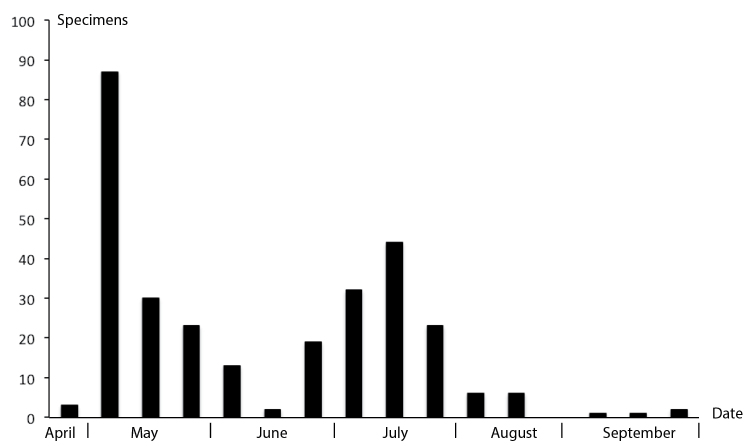
Histogram showing seasonal activity of *Megaselia lucifrons* adults as indicated by SMTP catches. The dates represent Malaise trap emptying dates.

So far, the data are too sparse to support strong assertions with respect to habitat preferences of *lucifrons* group species, although many specimens examined were collected in Malaise traps placed in wet or forested habitats. For *Megaselia lucifrons* it appears that there are two generations per year (Fig. 8) while data are too scarce to allow any definite conclusions to be drawn for the other two species.

## Discussion

To encourage additional taxonomic and systematic investigations of *Megaselia*, we believe it is important to study its phylogenetic structure so that it can be broken down into smaller, natural groups suitable for more detailed study. However, it will be some time before a clear picture of the deep phylogenetic structure of the entire genus will emerge, whose sufficiently broad sampling of lineages supports a stable subgeneric classification of the genus. Meanwhile, we think it will be productive to successively carve out and name monophyletic clades as informal species groups, which can later be revised in scope or renamed as knowledge increases. It is in this spirit we here define the *lucifrons* group.

Species of the *lucifrons* group have a wide and spinose labellum, which is quite uncommon for species of *Megaselia* of this small size (1.5–2.0 mm). The labellum is wide, more specifically, at least as wide as half of the frons (distance from middle of frons to dorsal frontalorbital seta). Costal ciliae are short (i.e. shorter than the third costal sector) which is characteristic for the group. The proctiger hair on the male genitalia in all three species of the *lucifrons* group is strong compared with hairs on the cercus (Fig. 5). The base of the left-arm projection in the male phallus is clearly sclerotized (Fig. 3c). This is not found in any other group within *Megaselia* with pilose anepisternum and is a likely synapomorphy for the *Megaselia lucifrons* group. In species similar to *Megaselia lata* (anepisternum without hairs), this sclerotization of the base of the left arm projection is also found, however the left arm projection itself is not present.

Disney formally synonymized *Megaselia lucifrons* and *Megaselia subnitida* in 1988 based on external morphological characters but without close examination of the male genitalia (Disney 1988). He had indicated previously that he regarded the differences between the two species described in the literature (Schmitz 1918) as intraspecific variation (Disney 1965). However, our molecular results show that *Megaselia subnitida* is genetically quite distinct from *Megaselia lucifrons*. The COI genetic distance is 15%, which is far above the threshold usually considered as indicating species-level differentiation (e.g., Stålstedt et al. 2013). This essentially confirms Schmitz’s original view on the species. Schmitz described morphological differences between the species in the wing venation (the fourth longitudinal vein is less bent in *Megaselia subnitida*), the surface and shape of the frons (*Megaselia lucifrons* has a shiny frons whereas *Megaselia subnitida* has a matte, slightly broader frons) and body size (*Megaselia subnitida* is the larger species). Aided by the molecular data, we were also able to identify additional differences in the male genitalia and the coloration of the hind legs.

Separation of species in *Megaselia* has been based primarily on differences in external morphological characters. Our results indicate that even slight variations in morphology, such as wing proportions and body size, can be good indicators for separating species. It is also clear that there are more morphological characters in the male genitalia than have been used previously for species identification. One reason that male genitalia characters remain poorly explored by phorid taxonomists is that many of the relevant characters are not possible to study when using the generally accepted slide mounting technique described by Disney (1983). Separation from the rest of the body and dissection of the genitalia is recommended to allow the study of these morphological characters (Nakayama 2012).

Substantial work remains to be completed before we can understand phylogenetic relationships within the genus *Megaselia* and address its relationships with other genera in the family. In the near future, we hope to contribute more broadly to resolving these problems with molecular methods (Häggqvist et al. in prep.). Until then, we need to develop methods that allow us to productively contribute small pieces to this puzzle. Our results argue for attempts to define and circumscribe monophyletic lineages of *Megaselia* based on morphological and molecular data as one of these methods.

## Taxonomy

### *Megaselia lucifrons* group

**Differential diagnosis.** Species of this group share the following features with the *lata* group (sensu stricto): The labellum is wide and spinose. When it is not expanded, such that pseudotracheae are visible, its width is between 130 and 170% of the half-width of the frons. The costal vein is almost half of the total wing length and the first costal sector is larger than the second and third together (Fig. 3a). The costal ciliae are short (i.e. shorter than the third costal sector). The notopleuron bears three equally strong setae, at approximately equal distance to each other. The proctiger hair on the male genitalia is strong compared with hairs on the cercus (Fig. 5). The base of the left-arm projection in the male phallus is clearly sclerotized (Fig. 3c).

Unlike the *lata* group (sensu stricto), the hind femur is lighter proximally in males of *Megaselia lucifrons* and *Megaselia albalucifrons*. It is unclear whether this is a ground-plan feature of the group, since the light femoral patch is lacking in males of *Megaselia subnitida*. However, the fact that the patch is present in the closely related *Megaselia lucifrons* suggests that the lack in *Megaselia subnitida* is secondary.

The anepisternum in all species of the group is pilose but without the setae found in some members of the subgenus *Aphiochaeta*.

**Remarks.** Three species are known in the group. They have been recorded from several European countries and likely have a Western Palaearctic or Palaearctic distribution. Since the diversity of *Megaselia* is poorly known in general, it seems likely that further species belonging to the group will be discovered. Females belonging to the *lucifrons* group can be recognized by the combination of a large labellum, characters in the wings and the pilose anepisternum, but we are not yet able to confidently identify them to species. The morphological diagnoses below refer, therefore, exclusively to males. The biology of the group as well as the relationships to other lineages in *Megaselia* is unknown (but see Fig. 1).

### Key to the males of the species

**Table:** 

1	Hind femur uniformly brown	***Megaselia subnitida***
–	Hind femur with triangular-shaped lighter patch at the base (Fig. 3b)	**2**
2	Left lobe of the hypandrium broad (Fig. 6a), triangular-shaped light patch at the base of the hind femur clearly defined	***Megaselia lucifrons***
–	Left lobe of hypandrium narrow (Fig. 6c), triangular-shaped light patch at the base of the hind femur weak	***Megaselia albalucifrons* sp. n.**

### Description of species

#### Megaselia lucifrons

Taxon classificationAnimaliaDipteraPhoridae

(Schmitz, 1918)

Aphiochaeta lucifrons Schmitz, 1918

##### Material examined.

Lectotype: male, pinned, Netherlands, Baaksem, Holl. Limbg. 11. VIII. 1915, coll. Schmitz (ZFMK).

##### Swedish material.

Suppl. material 1.

##### Differential diagnosis.

Males are easily distinguished from *Megaselia subnitida* by the triangular-shaped lighter patch on the base of the hind femur. *Megaselia lucifrons* also has a shiny frons (hence its name), whereas the frons is matte in *Megaselia subnitida*. Males can be distinguished from *Megaselia albalucifrons* by the broader left lobe of the hypandrium (Fig. 6a). The lobe is also darker in *Megaselia lucifrons* than in *Megaselia albalucifrons*. The left side of the epandrium has long thin hairs at the lower apical part that are typical for the species (Fig. 5a).

##### Distribution.

*Megaselia lucifrons* is widely distributed in Sweden and has been found in all 29 SMTP traps from which we have studied material. The species is also reported from many other European countries (e. g. Prescher et al. 2002; Brenner 2004; Schmitz 1928; Durska et al. 2010; Schmitz 1934; Bonet et al. 2011), however, for findings after 1988, when *Megaselia subnitida* was synonymized with *Megaselia lucifrons*, it is often impossible to determine from the literature alone whether the records refer to *Megaselia lucifrons* or *Megaselia subnitida*.

##### Biology.

Material from the SMTP shows that *Megaselia lucifrons* likely has two generations per year (Fig. 8), alternatively the flies overwinter as adults, as has been found for a number of other *Megaselia* species (Disney 1994). *Megaselia lucifrons* is found in different kinds of habitats from the SMTP, often in connection to trees or forest (Suppl. material 1).

#### Megaselia subnitida

Taxon classificationAnimaliaDipteraPhoridae

(Lundbeck, 1920) reinstated

Aphiochaeta subnitida Lundbeck, 1920
Megaselia subnitida Schmitz, 1927
Megaselia lucifrons Disney, 1988

##### Material examined.

Holotype: male, slide mounted, Denmark, Holte, Suserup Skov at Sorø, 6. VIII. 1918 (leg. Th. Mortensen) (ZMUC).

##### Swedish material.

Suppl. material 1.

##### Differential diagnosis.

Males of *Megaselia subnitida* are distinguished from males of the other two species in the group by the uniformly colored hind femur and the broader hypandrial left lobe (Fig. 6b). The frons is also less glabrous than in *Megaselia lucifrons* and *Megaselia albalucifrons*. Males of *Megaselia subnitida* are generally larger in size than both *Megaselia lucifrons* and *Megaselia albalucifrons* (Table 1).

##### Distribution.

*Megaselia subnitida* is widely distributed in Sweden, and is usually found together with *Megaselia lucifrons* (Fig. 7). Schmitz (1928) listed *Megaselia subnitida* from Sweden, Finland, Denmark, Germany and Poland. In the more recent literature, after Disney synonymized the species with *Megaselia lucifrons* (Disney, 1988), separate records for *Megaselia subnitida* are not reported.

##### Biology.

*Megaselia subnitida* appears in different habitats, often in or in proximity to a forest, from May to September in the SMTP material (Suppl. material 1).

##### Remarks.

In Disney’s key (1989), *Megaselia subnitida* runs to couplet 104, together with *Megaselia lucifrons*.

#### Megaselia albalucifrons sp. n.

Taxon classificationAnimaliaDipteraPhoridae

http://zoobank.org/491B368D-3065-403A-919F-A2AB5FE08ABE

##### Material examined.

Holotype male, in alcohol, (LA214) SWEDEN, Sm, Älmhults kommun, Stenbrohult, Djäknabygds bokbacke, Heath with old beeches. N56°36.548' E14°11.583' (=TrapID 24) 30.iv.-31-v.2005 (=coll. event ID 1672) Leg. Swedish Malaise Trap Project (Swedish Museum of Natural History).

Paratypes, 4 males, same as holotype (LA207, LA212, LA215, LA216).

##### Differential diagnosis.

The habitus (Fig. 2) is very similar to *Megaselia lucifrons*, but the species can be distinguished by the narrower and more lightly colored hypandrial left lobe (Fig. 6c). The proctiger hair is also slightly stronger than in *Megaselia lucifrons* (Fig. 5). In the phallus, the tip of the truncus (Fig. 3c) is more rounded in *Megaselia albalucifrons* than in both *Megaselia lucifrons* and *Megaselia subnitida*.

##### Description.

Male: Frons dark brown and shiny with about 70 hairs, about as broad as long. Postpedicels brown. Palps light yellow with 6–8 setae. Labellum broad, light yellow as palps, densely covered with spinules. Anepisternum with 10–18 hairs. Thorax brown. Three notopleural setae, without cleft in front of them. Scutellum with anterior pair of hairs and posterior pair of setae. Abdominal tergites brown with hairs distally and laterally, especially T6 with long, strong hairs at the rear margin. Venter brown with hairs on segment 3–6. Hypopygium with brown epandrium with fine hairs on both sides, but not dorsally, and longer hairs at the lower margin (Fig. 5, left side of epandrium). Pale yellow to light brown anal tube, cercus with 14 downbent hairs and the somewhat downbent proctiger with strong pair of upbent hairs at the tip (Fig. 5). Hypandrium brown, except for pale left lobe. Legs colored as follows: Fore and middle legs yellow or brownish yellow, hind legs with coxae yellow and femur to tarsae brown. Hind femur with lighter, triangular shaped patch at the base. Wings (Fig. 3a) 1.3–1.6 mm long. Costal index 0.40–0.47. Costal ratios 4.0–4.4 : 1.5–1.8 : 1. Costal cilia (of section 3) 0.05–0.07 mm long. With 3 axillary setae, all about twice as long as costal cilia. A minute hair is situated at the base of vein 3. Veins light brown or brown. Wing membrane tinged light brown or brown (visible to the naked eye against a white background). Haltere knob light yellow, almost white.

##### Etymology.

The name is derived from the Latin word “albus” (white, pale), referring to the left lobe of the hypandrium, which is pale in comparison to that of *Megaselia lucifrons*.

##### Distribution and ecology.

*Megaselia albalucifrons* is found in Southern and central Sweden (Fig. 7), so far no specimens have been found from traps in Northern Sweden.

##### Remarks.

*Megaselia albalucifrons* runs to couplet 104 in Disney’s key (1989), together with *Megaselia lucifrons*.

## Supplementary Material

XML Treatment for Megaselia lucifrons

XML Treatment for Megaselia subnitida

XML Treatment for Megaselia albalucifrons

## Supplementary materials

Supplementary material 1 Data type: species data Explanation note: All specimens, except LA208 and LA209, which were caught by hand, come from the Swedish Malaise Trap Project (SMTP). All specimens are deposited in the collection of the Swedish Museum of Natural History. This dataset is made available under the Open Database License (http://opendatacommons.org/licenses/odbl/1.0/). The Open Database License (ODbL) is a license agreement intended to allow users to freely share, modify, and use this Dataset while maintaining this same freedom for others, provided that the original source and author(s) are credited.Sibylle Häggqvist, Sven Olof Ulefors, Fredrik Ronquist

Supplementary material 2 Figure S1. Majority rule consensus tree from a Bayesian analysis of lucifrons group relationships based on COI data Data type: molecular data Explanation note: *Megaselia lata* and *Megaselia hibernans* were used as outgroups. Numbers on branches are posterior probabilities (PP, in percent). PP values are not given for branches inside the terminal clusters corresponding to species. The scale bar represents nucleotide substitutions per site. This dataset is made available under the Open Database License (http://opendatacommons.org/licenses/odbl/1.0/). The Open Database License (ODbL) is a license agreement intended to allow users to freely share, modify, and use this Dataset while maintaining this same freedom for others, provided that the original source and author(s) are credited.Sibylle Häggqvist, Sven Olof Ulefors, Fredrik Ronquist

Supplementary material 3 Figure S2. Majority rule consensus tree from a Bayesian analysis of lucifrons group relationships based on 28S D2 data Data type: molecular data Explanation note: *Megaselia hibernans* were used as outgroups. Numbers on branches are posterior probabilities (PP, in percent). PP values are not given for branches inside the terminal clusters corresponding to species. The scale bar represents nucleotide substitutions per site. This dataset is made available under the Open Database License (http://opendatacommons.org/licenses/odbl/1.0/). The Open Database License (ODbL) is a license agreement intended to allow users to freely share, modify, and use this Dataset while maintaining this same freedom for others, provided that the original source and author(s) are credited.Sibylle Häggqvist, Sven Olof Ulefors, Fredrik Ronquist
