# Supplementary figures and images for: A new species group in Megaselia, the lucifrons group, with description of a new species (Diptera, Phoridae)
Source: Zookeys. 2015 Jul 6;(512):89–108. doi: 10.3897/zookeys.512.9494 (PMC4523756; doi:10.3897/zookeys.512.9494)

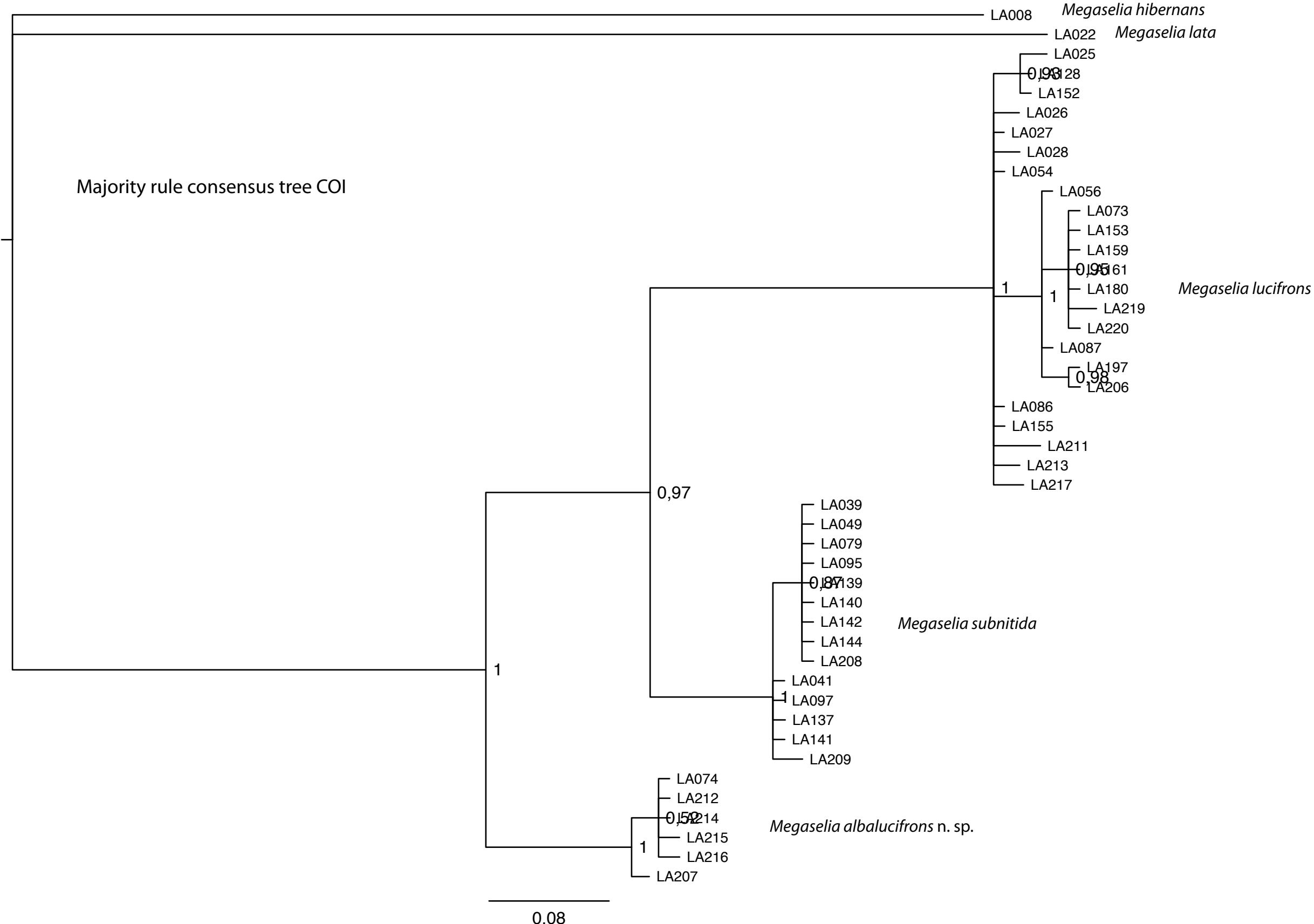

Supplement: Supplementary material 2 — Figure S1. Majority rule consensus tree from a Bayesian analysis of lucifrons group relationships based on COI data [file zookeys-512-089-s002.pdf]

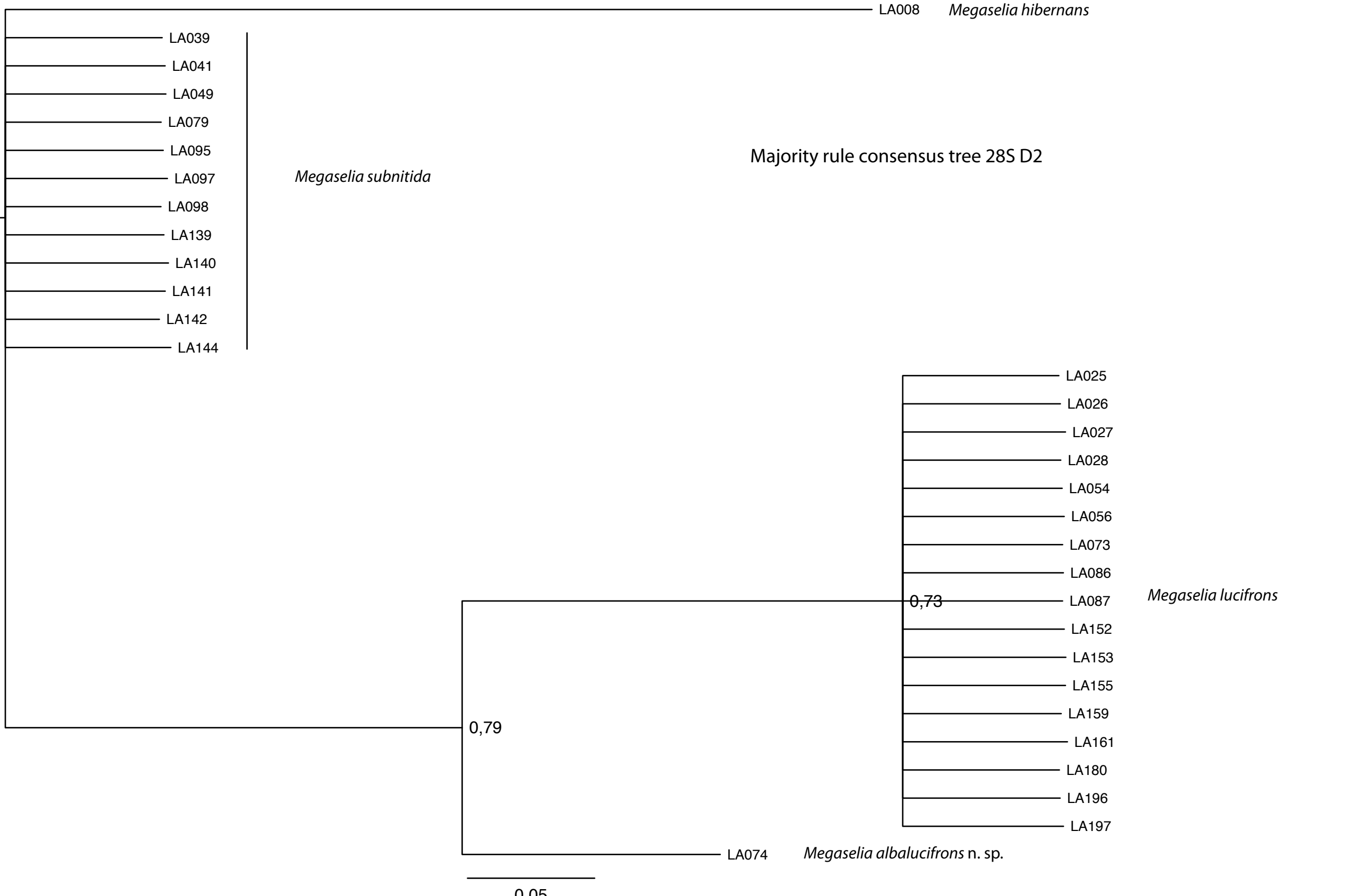

Supplement: Supplementary material 3 — Figure S2. Majority rule consensus tree from a Bayesian analysis of lucifrons group relationships based on 28S D2 data [file zookeys-512-089-s003.pdf]
